# UK Supreme Court Ruling: A step backwards for transgender inclusionary healthcare?

**DOI:** 10.1016/j.fhj.2026.100504

**Published:** 2026-01-29

**Authors:** Simrit Braich

**Affiliations:** Lancashire Teaching Hospitals, Preston, United Kingdom

On 16 April 2024, the UK Supreme Court delivered a ruling that marks a significant shift in how legal definitions are interpreted within the Equality Act 2010. The Court concluded that the words ‘woman’ and ‘sex’ refer strictly to biological definitions, meaning that individuals with a Gender Recognition Certificate (GRC) identifying them as female do not meet the Act’s criteria for ‘woman’.^1^ Previously, under the Gender Recognition Act 2004, individuals were treated in accordance with their legal sex, as stated on their GRC, including within healthcare settings.^2^ Gender reassignment is a protected characteristic, therefore discriminating against someone for being transgender is unlawful. However, the ruling adds complexity to how this plays out in practice. Single-sex spaces such as women’s shelters, changing rooms and hospital wards are now legally permitted to exclude trans women, provided that it is justifiable and proportionate.^1^ This creates a paradox: protected *as a woman*, but not fully treated as one. It also raises the question of how transgender men will be defined and treated within these spaces.

In early September 2025, the Equality and Human Rights Commission (EHRC) submitted an updated draft Code of Practice for services, public functions and associations; this provides guidance on how to deliver public services in accordance with the Equality Act. It currently awaits approval to be laid before parliament and, following a mandatory 40-day scrutiny period, the government will decide whether to accept or reject the proposed changes.^3^ This decision is likely to have far-reaching effects on the transgender community, including access and experiences within healthcare.

The British Medical Association (BMA) acknowledges the significant inequalities faced by trans and non-binary people in accessing health services.^4^ A demographic who already experience worse access to care, poorer treatment and less desirable health outcomes. The Supreme Court ruling has not accounted for intersex variation, gender identity, gender dysphoria or the nuance of transgender experiences. If the law does not recognise or respect trans and intersex individuals, how can they feel safe in the institutions that are meant to protect them?

Avoidance of healthcare is widespread among trans individuals and is driven not only by anxiety, but also by real fears of discrimination.^5^ Adhering to a rigid sex/gender binary excludes those who don’t conform, often making them feel ostracised and further limiting access to inclusive medical care.^6^ The extension of a non-inclusive code of practice into healthcare policy will make it harder for transgender individuals to exist without constraint.

Previously, NHS policy allowed transgender individuals to be accommodated according to their gender expression, based on names, pronouns and appearance. Following the ruling, this approach was challenged by interim guidance from the EHRC, published on 25 April 2025 and stating that, in some cases, trans women and trans men could be excluded from both women’s and men’s facilities.^7^ Delivered in haste, with an unclear message, it created confusion and apprehension. The NHS was also warned that it would be ‘pursued’ by the organisation if it failed to revise its policies to align with the new interim guidance; however, there was no further commentary on what that would entail.^8^ Contrary to this, multiple NHS trusts^9^, ^10^, ^11^ released statements that publicly affirmed their commitment to inclusivity and supporting gender-diverse/trans colleagues and patients under the Equality Act – ‘We will continue to see and treat trans people with the dignity and respect they both deserve and can expect under the current UK law’.

In a U-turn of events, the EHRC withdrew the interim guidance 6 months after publication. It previously drew strong criticism from trans rights groups for risking discriminatory exclusion from major areas of public life.^3^^,^^12^ Concerns have been raised by 18 UN independent experts about the EHRC’s handling of the updated code of practice, including warnings about the legality, cost and compatibility with basic rights. Recently, Amnesty International UK reported the EHRC to the Global Alliance of National Human Rights Institutions (GANHRI) for violating the Paris Principles, which set the minimum standards for credible and effective national human rights institutions.^13^

Discrimination in healthcare can manifest in numerous ways, including denial of services, misgendering and inappropriate questioning.^14^ In practice, withholding access to gender-affirming spaces amounts to ‘outing’ people. In January 2024, Trans Actual responded to health secretary Wes Streeting’s suggestion of transgender-only hospital wards, stating that ‘forcing someone to stay in a segregated trans-specific area will not protect their dignity, does not indicate respect for them as a human being, and may act to the detriment of the care they receive’.^15^ It’s an example of how those shaping healthcare policy lack real understanding of the wider impact. When there is compelling evidence that gender-affirming care improves mental health and wellbeing and reduces suicide risk,^16^ it raises critical questions: Should patients or clinicians accept anything less than services that are tailored and appropriate? Would a lack of suitable provision be acceptable in any other context?

It is important to acknowledge that, to date, the law remains unchanged and this has not proven detrimental. Research consistently finds that inclusive provision does not increase harm or victimisation of others. In contrast, the Gendered Spaces Review highlights how the Supreme Court ruling and EHRC interim guidance have caused significant lived distress for both trans women and cis women, who have been incorrectly misgendered.^17^ Pressure on the government to make a careful decision comes from more than just human rights activists and transgender allies. Dozens of backbench MPs have written to the business secretary to express significant concern from industries who would be stuck in a legal minefield, ‘the unresolved legal contradiction between the potential for being sued for challenging someone’s gender versus being sued for failing not to’ and acknowledging the discomfort of their staff who aren’t employed to police gender.^18^

Regardless of how the government rules on the updated code of practice, the story is far from over – it marks only the beginning of a broader debate. Challenges should be expected both in parliament and before the ECHR, whether that be by gender-critical groups or equality-driven advocates. Healthcare must serve everyone, not just the majority. This principle was upheld by resident doctors, who opposed the Supreme Court ruling as scientifically illiterate and reductive at the 2025 BMA conference.^19^ As the NHS prepares for discriminatory policy change, now is the time for healthcare professionals to act. Transgender individuals should not just be heard on the sidelines in protest, but actively influencing the frameworks that impact their lives. In practice, this means holding responsible bodies accountable for engaging with trans-led specialist interest groups, opposing exclusionary practices, and recognising diversity as a strength. Dignity, respect and access to appropriate healthcare are not optional, they are fundamental rights. The real question is not about definitions, but why systems remain resistant to inclusivity.

## Acknowledgments

I would like to thank Mafaal Faal-Mason for his ongoing support and discussion in relation to this piece.

## Declaration of generative AI and AI-assisted technologies in the writing process

During the preparation of this work, the author used ChatGPT in order to help draft the reference list. The author provided the relevant webpages, titles, authors and DOIs, and the tool produced draft references. All references were subsequently checked, corrected and completed by the author, including adding access dates. After using this tool/service, the author then reviewed and edited the content as needed and takes full responsibility for the content of the published article.

## Funding

This research did not receive any specific grant from funding agencies in the public, commercial or not-for-profit sectors.

## CRediT authorship contribution statement

**Simrit Braich:** Writing – review & editing, Writing – original draft, Conceptualization.

## Declaration of competing interest

The authors declare that they have no known competing financial interests or personal relationships that could have appeared to influence the work reported in this paper.

## References

[1] UK Supreme Court rules legal definition of a woman is based on biological sex. BBC News; 2025. https://www.bbc.co.uk/news/live/cvgq9ejql39t. Accessed 13 November 2025.

[2] TransActual. The Gender Recognition Act 2004; 2025. https://transactual.org.uk/the-gender-recognition-act-2004. Accessed 21 April 2025.

[3] Bevan Brittan LLP. EHRC urges government to act and withdraws its interim update. 2025 https://www.bevanbrittan.com/insights/articles/2025/ehrc-urges-government-to-act-and-withdraws-its-interim-update/. Accessed 13 November 2025.

[4] British Medical Association. Inclusive care of trans and non-binary patients. https://www.bma.org.uk/advice-and-support/equality-and-diversity-guidance/lgbtplus-equality-in-medicine/inclusive-care-of-trans-and-non-binary-patients. Accessed 13 November 2025.

[5] Hobster K., McLuskey J. (2020). Transgender patients’ experiences of health care. Br J Nurs.

[6] Morrison T., Dinno A., Salmon T. (2021). The erasure of intersex, transgender, nonbinary, and agender experiences through misuse of sex and gender in health research. Am J Epidemiol.

[7] A Bond, C Leckey, C Soakell, Equality Watchdog Guidance on Singlesex Facilities. London: Lewis Silkin LLP; 2025. https://www.lewissilkin.com/insights/2025/04/26/equality-watchdog-guidance-on-single-sex-facilities. [Accessed 13 November 2025].

[8] The Independent NHS to be pursued if gender policies on single-sex wards don’t change, warns equalities watchdog. 2025. https://www.independent.co.uk/news/uk/politics/trans-women-nhs-supreme-court-ruling-b2734976.html Accessed 13 November 2025

[9] Leeds and York Partnership NHS Foundation Trust. Response to Supreme Court ruling on the Equality Act. 2025. https://www.leedsandyorkpft.nhs.uk/news/articles/response-to-supreme-court-ruling-equality-act/leedsandyorkpft.nhs.uk Accessed 13 November 2025

[10] Chesterfield Royal Hospital NHS Foundation Trust. UK Supreme Court ruling. 2025. https://www.chesterfieldroyal.nhs.uk/latest-news/uk-supreme-court-ruling Accessed 13 November 2025.

[11] Nottinghamshire Healthcare NHS Foundation Trust. UK Supreme Court ruling on legal definition of a woman –16 April 2025. 2025. https://www.nottinghamshirehealthcare.nhs.uk/latest-news/uk-supreme-court-ruling-on-legal-definition-of-a-woman-16-april-2025-8104/ Accessed 13 November 2025.

[12] Geneva Council. EHRC withdraws interim guidance on transgender exclusion: a critical moment for UK human rights. 2025 https://genevacouncil.com/ehrc-withdraws-interim-guidance-on-transgender-exclusion-a-critical-moment-for-uk-human-rights/ Accessed 13 November 2025.

[13] Amnesty International UK. UK: Amnesty and other key rights organisations challenge the Equality and Human Rights Commission for violating its human rights obligations. 2025. https://www.amnesty.org.uk/press-releases/uk-amnesty-and-other-key-rights-organisations-challenge-equality-and-human-rights Accessed 13 November 2025.

[14] Mikulak M., Ryan S., Ma R. (2021). Health professionals’ identified barriers to trans health care: a qualitative interview study. Br J Gen Pract.

[15] TransActual Editor. Statement on Wes Streeting’s comments about trans-specific hospital wards. TransActual; 2024. https://transactual.org.uk/blog/2024/01/30/statement-on-wes-streetings-comments-about-trans-specific-hospital-wards/ Accessed 13 November 2025.

[16] Lawson Z., Davies S., Harmon S. (2023). A human rights based approach to transgender and gender expansive health. Clin Psychol Forum.

[17] TransActual. Gendered spaces and the impact of the Supreme Court judgement/EHRC Interim update on Trans, Intersex and GenderNonConforming adults. 2025. https://transactual.org.uk/wp-content/uploads/Gendered-Spaces-Review-Final.pdf Accessed 13 November 2025.

[18] Walker P. Dozens of Labour MPs warn of chaos for firms over gender recognition advice. The Guardian. 2025. https://www.theguardian.com/society/2025/oct/23/dozens-of-labour-mps-warn-of-chaos-for-firms-over-gender-recognition-advice Accessed 13 November 2025.

[19] BMA Residents. Attendees at the #RDconf last Saturday voted on an emergency motion to show their opposition to the Supreme Court ruling on [definition of a woman] 2025. https://x.com/BMAResidents/status/1917186510526296384. Accessed 13 November 2025.

